# Extramotor Damage Is Associated with Cognition in Primary Lateral Sclerosis Patients

**DOI:** 10.1371/journal.pone.0082017

**Published:** 2013-12-05

**Authors:** Elisa Canu, Federica Agosta, Sebastiano Galantucci, Adriano Chiò, Nilo Riva, Vincenzo Silani, Andrea Falini, Giancarlo Comi, Massimo Filippi

**Affiliations:** 1 Neuroimaging Research Unit, Institute of Experimental Neurology, Division of Neuroscience, San Raffaele Scientific Institute, Vita-Salute San Raffaele University, Milan, Italy; 2 Department of Neurology, Institute of Experimental Neurology, Division of Neuroscience, San Raffaele Scientific Institute, Vita-Salute San Raffaele University, Milan, Italy; 3 Department of Neuroradiology, CERMAC, San Raffaele Scientific Institute, Vita-Salute San Raffaele University, Milan, Italy; 4 ‘Rita Levi Montalcini’ Department of Neuroscience, University of Torino, Torino, Italy; 5 Department of Neurology and Laboratory of Neuroscience, IRCCS Istituto Auxologico Italiano - Department of Pathophysiology and Tranplantation, “Dino Ferrari” Center, Università degli Studi di Milano, Milan, Italy; University of Ulm, Germany

## Abstract

**Objectives:**

This is a cross-sectional study aimed at investigating cognitive performances in patients with primary lateral sclerosis (PLS) and using diffusion tensor (DT) magnetic resonance imaging (MRI) to determine the topographical distribution of microstructural white matter (WM) damage in patients with or without cognitive deficits.

**Methods:**

DT MRI scans were obtained from 21 PLS patients and 35 age- and sex-matched healthy controls. All PLS patients underwent a comprehensive neuropsychological battery. Tract-based-spatial-statistics (TBSS) was used to perform a whole-brain voxel-wise analysis of fractional anisotropy (FA), axial, radial (radD) and mean diffusivity (MD).

**Results:**

Ten PLS patients had abnormal scores in at least one neuropsychological test (PLS with cognitive deficits, PLS-cd). Compared with healthy controls and cognitively unimpaired PLS patients (PLS-cu), PLS-cd cases showed decreased FA and increased MD and radD in the corticospinal tract (CST), corpus callosum, brainstem, anterior limb of internal capsule, superior and inferior longitudinal fasciculi, fornix, thalamic radiations, and parietal lobes, bilaterally. Compared with healthy controls, PLS-cd patients showed further decreased FA and increased radD in the cerebellar WM, bilaterally. Compared with controls, PLS-cu patients showed decreased FA in the mid-body of corpus callosum. In PLS, executive and language test scores correlated with WM damage.

**Conclusions:**

This is the first study evaluating the relationship between cognitive performance and WM tract damage in PLS patients. PLS can be associated with a multi-domain cognitive impairment. WM damage to interhemispheric, limbic and major associative WM tracts seem to be the structural correlate of cognitive abnormalities in these patients.

## Introduction

Primary lateral sclerosis (PLS) is a rare, progressive disorder characterised by an isolated degeneration of the upper motor neurons (UMN) in the absence of lower motor neuron (LMN) signs [1]. Longitudinal studies have estimated that 2–5% of patients seen in adult neuromuscular clinics are diagnosed with PLS [1], [2]. PLS patients present with an insidious onset of a symmetric, spastic paresis, usually beginning in the lower extremities [1]. Compared with amyotrophic lateral sclerosis (ALS), PLS has a slower rate of progression and a more benign prognosis with a survival of more than 10 years from symptom onset [2].

A detectable degree of cognitive involvement, which can vary in magnitude, appears in many patients with ALS [3]. In ALS, deficits at tests of executive functions are more commonly found, with verbal fluency as the most affected one, followed by set shifting, cognitive inhibition and selective attention [4]. The language domain is also affected in ALS (especially confrontation naming), and memory deficits, although less studied, have been reported in encoding rather than in retrieval [4]. A few studies so far have investigated the cognitive status of PLS patients and findings are controversial with some authors reporting no cognitive abnormalities [1] and others describing the occurrence of cognitive impairment which extends beyond the executive functions to also involve memory and language [2], [5]–[8].

Diffusion tensor (DT) magnetic resonance imaging (MRI) is currently unrivalled as a neuroimaging marker of UMN involvement and has the potential to provide an objective *in vivo* assessment of the extramotor brain damage in ALS and other motor neuron disorders (MND) [9]. White matter (WM) abnormalities in PLS have been classically reported in the corticospinal tract (CST) and corpus callosum (CC) [10]–[14]. Nevertheless, DT MRI studies have shown that extramotor regions are not spared in PLS, including frontal, temporal and parietal WM areas [10]–[14].

Aim of this study was to investigate cognitive performances in patients with PLS and to use DT MRI to determine the topographical distribution of microstructural WM damage in patients with or without cognitive deficits.

## Methods

### Subjects

Patients with a clinically definite diagnosis of PLS [1] were recruited prospectively between April 2010 and July 2011. All patients had no evidence of acute or chronic denervation on repeated electromyographical examinations and had their symptoms for at least 3 years. To be eligible, subjects had to meet the following criteria: no family history of MND; no clinical diagnosis of frontotemporal dementia (FTD) [15]; age at onset ≥ 40 years and no mutations of major genes related to hereditary spastic paraparesis (i.e., SPG3A, SPG4, SPG6, SPG7 and SPG20); no any other major systemic, psychiatric or neurological illnesses; no history of substance abuse; and no other causes of focal or diffuse brain damage, including strokes, lacunae and other evidence of cerebrovascular disease at routine MRI scans. Within 48 hours from MRI, functional status was assessed using the ALS Functional Rating Scale (ALSFRS-r) [16] and clinical UMN involvement was graded according to the UMN score [17]. The rate of disease progression at study entry was calculated as follows: (48 – ALSFRS-r score)/ time from symptom onset [18].

Twenty-six PLS patients were recruited [10]. Five patients were excluded because of unwillingness to perform the cognitive evaluation (age 66±10 years; 60% women; 100% spinal onset; disease duration 139±32 months; ALSFRS-r 39±2; UMN score 15±0.9; disease progression rate 0.07±0.02). Twenty-one patients, who were assessed using a comprehensive neuropsychological battery and MRI scan, were included in the current study (Table 1). Thirty-five healthy controls were recruited among spouses of patients and by word of mouth; they underwent a neurological evaluation which assessed neurological and other medical symptoms, family history for neurological and major mental conditions, and global cognition using the Mini Mental State Examination (MMSE). Participants were included in the study only if the assessment was normal (Table 1). The study was approved by the Ethical Committee of San Raffaele Scientific Institute, Milan, Italy. All subjects provided written informed consent before enrolment.

**Table 1:** Sociodemographic and clinical features of the three study groups.

	HC	PLS-cu	PLS-cd	p^1^	p^2^
Number	35	11	10		
Age at MRI [years]	63.9±8.9 (43–79)	60.1±9.7 (43–70)	63.6±5.6 (55–72)	0.647	0.622
Gender (women)	19 (54%)	6 (55%)	5 (50%)	0.969	1.000.
Education [years]	11.0±2.3 (8–13)	9.6±4.2 (2–18)	8.9±3.9 (5–18)	0.153	0.478
MMSE (co: 24)	29.7±0.5 (29–30)	27.6±1.5 (26–30)	25.4±3.6 (16–28)*	0.018	0.090
Bulbar symptoms at the onset	-	2 (18%)	0 (0%)	-	0.476
Disease duration [months]	-	88.4±56.1 (48–236)	98.4±61.2 (38–247)	-	0.778
ALSFRS-r	-	35.3±6.4 (24–42)	36.5±6.8 (22–41)	-	0.755
UMN score	-	12.9±2.0 (10–16)	13.9±1.3 (12–16)	-	0.415
Disease progression rate [ALSFRS-r score/months]	-	0.19±0.12 (0.03–0.4)	0.15±0.10 (0.04–0.4)	-	0.360
WMH load [ml]	0.7±1.0 (0–5)	0.3±0.4 (0–1)	0.6±1.0 (0–3)	0.452	0.519

Values are mean ± standard deviation [range] or number (%). P^1^ =  differences between all groups; P^2^ = differences between patient groups. *p<0.05 compared with healthy controls. Group differences in categorial variables (i.e., gender and onset type) were assessed using the Fisher Exact test. Continuous variables (i.e., age, WMH load, disease duration, ALSFRS-r, UMN score, and disease progression rate) were compared using the Kruskal-Wallis or the Mann-Whitney U-test. Disease progression rate = (48-ALSFRS-r score)/time from symptom onset. Abbreviations: ALSFRS-r =  ALS Functional Rating scale-revised; co =  cut-off; MMSE =  mini mental state examination; PLS-cd =  primary lateral sclerosis with cognitive deficits; PLS-cu =  cognitively unimpaired primary lateral sclerosis; UMN score =  upper motor neuron score; WMH =  white matter hyperintensity.

### Neuropsychological assessment

A comprehensive neuropsychological battery was administered to all patients by an experienced neuropsychologist who was unaware of MRI results and investigated global cognition with the MMSE [19]; executive functions with the semantic and phonemic fluency [20], fluency indices (controlling for individual variations in motor disabilities) [21], Weigl’s test [22],Wisconsin Card Sorting Test (WCST) [23], digit span backward [24], and Cognitive Estimation Test (CET) [25]; reasoning and abstraction abilities with the Raven coloured progressive matrices [26]; verbal memory with the digit span forward [24] and the Rey’s word list [27]; and language with the BADA oral naming of objects and actions [28]. Mood and behaviour were assessed using the Hamilton Depression Rating Scale (HDRS) [29] and the Frontal Behavioural Inventory (FBI, administered to the caregivers) [30]. Scores on neuropsychological tests were age-, sex-, and education-corrected by using related normative values. We defined as PLS with cognitive deficits (PLS-cd) those patients who performed below the 5^th^ percentile in at least one cognitive test within the executive, memory and/or language domains, and as cognitively unimpaired (PLS-cu), PLS patients who performed within the normal range in all considered domains.

### MRI acquisition

Brain MRI scans were obtained using a 3.0 T scanner (Intera, Philips Medical Systems, Best, The Netherlands). The following sequences were acquired from all subjects: (i) T2-weighted spin echo (SE) (repetition time [TR] = 3500 ms, echo time [TE] = 85 ms, echo train length = 15, flip angle = 90°, 22 contiguous, 5 mm-thick axial slices with a matrix size = 512×512, field of view [FOV] = 230×184 mm^2^); (ii) fluid-attenuated inversion recovery (FLAIR) (TR = 11000 ms, TE = 120 ms, flip angle = 90°, 22 contiguous, 5 mm-thick axial slices with a matrix size = 512×512, FOV = 230×230 mm^2^); (iii) 3D T1-weighted fast field echo (TR = 25 ms, TE = 4.6 ms, flip angle = 30°, 220 contiguous axial slices with voxel size = 0.89×0.89×0.8 mm, matrix size = 256×256, FOV = 230×182 mm^2^); and (iv) pulsed-gradient SE echo planar with sensitivity encoding (acceleration factor = 2.5, TR = 8986 ms, TE = 80 ms, 55 contiguous, 2.5 mm-thick axial slices, number of acquisitions = 2; after SENSE reconstruction, the matrix dimension of each slice was 256×256, with an in-plane pixel size of 0.94×0.94 mm and a FOV = 240×240 mm^2^) and with diffusion gradients applied in 32 non-collinear directions, using a gradient scheme which is standard on this system (gradient over-plus) and optimised to reduce echo time as much as possible. The b factor used was 1000 s/mm^2^. Fat saturation was performed to avoid chemical shift artefacts. All slices were positioned to run parallel to a line that joins the most infero-anterior and infero-posterior parts of the CC.

### MRI analysis

MRI analysis was performed by an experienced observer, blinded to subjects’ identity. WM hyperintensities (WMH), if any, were identified on T2-weighted and FLAIR scans. WMH load was measured using the Jim software package (Version 5.0, Xinapse Systems, Northants, UK, http://www.xinapse.com).

**DT MRI preprocessing.** DT MRI analysis was performed using the FMRIB software library (FSL) tools (http://www.fmrib.ox.ac.uk/fsl/fdt/index.html) and the JIM5 software. The diffusion-weighted data were skull-stripped using the Brain Extraction Tool (BET) implemented in FSL. Using FMRIB's Linear Image Registration Tool (FLIRT), the two diffusion-weighted scans were coregistered by applying the rigid transformation needed to correct for position between the two b_0_ images (T2-weighted, but not diffusion-weighted). The rotation component was also applied to diffusion-weighted directions. Eddy currents correction was performed using the JIM5 software. Then, the two acquisitions were concatenated. The DT was estimated on a voxel-by-voxel basis using DTIfit provided by the FMRIB Diffusion Toolbox. Maps of mean diffusivity (MD), fractional anisotropy (FA), axial diffusivity (axD) and radial diffusivity (radD) were obtained.

**Voxel-based analysis: TBSS.** TBSS version 1.2 (http://www.fmrib.ox.ac.uk/fsl/tbss/index.html) was used to perform the multi-subject DT MRI analysis. FA volumes were aligned to a target image using the following procedure: (i) the FA template in standard space (provided by FSL) was selected as the target image, (ii) the non-linear transformation that mapped each subject's FA to the target image was computed using the FMRIB's Non-linear Image Registration Tool (FNIRT), and (iii) the same transformation was used to align each subject's FA to the standard space. A mean FA image was then created by averaging the aligned individual FA images, and thinned to create a FA skeleton representing WM tracts common to all subjects. The FA skeleton was thresholded at a value of 0.2 to exclude voxels with low FA values, which are likely to include grey matter or cerebrospinal fluid. Individual MD, FA, axD and radD data were projected onto this common skeleton.

### Statistical analysis

**Demographic, clinical, cognitive and conventional MRI data.** Group differences in categorial variables were assessed using the Fisher Exact test. Continuous variables were compared using the Kruskal-Wallis or the Mann-Whitney U-test, as appropriate (SAS Release 9.1, SAS Institute, Cary, NC, USA; p value <0.05).

**TBSS: between-group comparisons.** DT MRI voxelwise statistics were performed using a permutation-based inference tool for nonparametric statistical thresholding (“randomise”, FSL [31]). The number of permutations was set at 5000 [31]. MD, FA, axD, and radD values within the skeleton were compared between groups adjusting for age. The between-group comparisons were thresholded at p<0.05, corrected for multiple comparisons at the cluster level using the threshold-free cluster enhancement (TFCE) option.

**TBSS: Relationships between cognitive features and WM microstructural damage.** To assess whether the DT MRI abnormalities were associated with cognition, regression models were run in FSL. Results were assessed at p<0.05, corrected for multiple comparisons at the cluster level using the TFCE option.

## Results

### Clinical, demographic and neuropsychological findings

Subject groups did not differ in terms of age at MRI, gender, education and WMH load (Table 1). There were 10 PLS-cd and 11 PLS-cu patients. Patient groups were similar in terms of ALSFRS-r score, UMN score, disease duration and disease progression rate (Table 1). Table 2 shows neuropsychological findings. One PLS-cd patient scored below the 5^th^ percentile in two executive tests (phonemic fluency and CET) and one language test (BADA oral naming), and has been defined as “cognitively impaired” according to the Strong consensus criteria [32]. In this case, phonemic fluency index was 30.2 suggesting that the fluency disturbances were pure and not affected by motor impairment. Two PLS-cd patients showed abnormal scores in one executive test (phonemic fluency or WCST - both WCST global score and inability to maintain the set) and one test assessing non-executive domains (memory/digit span or language/action naming). Three PLS-cd cases presented with a decline in one executive test only (two at the WCST and one at the semantic fluency), and four PLS-cd patients scored below the 5^th^ percentile in one non-executive cognitive test only (three: language/action naming; one: memory/Rey’s word list recall).

**Table 2:** Neuropsychological and behavioral data of PLS patients.

	PLS-cu	PLS-cd	p
**N**	11	10	
***Executive functions***			
WCST, global score (co: 90.5)	33.2±27.4 (8–78)	71.6±29.5 (18–102)	0.024
WCST, perseverative responses (co: 42.6)	11.4±11.6 (1–30)	19.9±11.7 (3–31)	0.302
Phonemic fluency (co: 17)	30.1±10.2 (17–52)	23.1±6.8 (16–33)	0.161
Phonemic fluency index	6.7±1.6 (4–9)	13.1±10.1 (5–31)	0.247
Semantic fluency (co: 25)	44.4±11.3 (32–62)	33.9±6.3 (19–39)	0.026
Semantic fluency index	4.6±1.2 (3–7)	5.6±1.7 (4–8)	0.487
Raven’s progressive matrices (co: 18)	30.2±4.6 (22–35)	28.7±5.6 (18–36)	0.437
Weigl test (co: 4.5)	12.6±1.9 (10–16)	10.6±3.2 (6–14)	0.246
Digit Span backward	4.5±0.9 (3–6)	3.9±1.1 (3–6)	0.190
Cognitive Estimation Test, total (co: 18)	13.0±2.3 (8–15)	14.2±4.2 (10–24)	0.884
***Memory***			
Digit span forward (co: 3.75)	5.9±1.4 (4–9)	5.2±0.9 (3–6)	0.245
Rey’s word list, imm. recall (co: 28.53 )	41.2±10.1 (31–62)	41.8±8.4 (34–58)	0.665
Rey’s word list, delay recall (co: 4.69)	9.0±3.3 (5–16)	8.8±2.6 (4–13)	0.962
***Language***			
BADA, oral object naming, errors (co: 2)	0.8±0.8 (0–2)	0.9±1.1 (0–3)	0.925
BADA, oral action naming, errors (co: 2)	1.0±0.8 (0–2)	3.1±2.3 (0–7)	0.040
***Mood***			
HDRS, (co: 7)	6.6±2.9 (2–10)	7.1±7.9 (1–24)	0.561
FBI, total (co: 26)	5.1±7.9 (0–24)	2.6±4.1 (0–9)	0.531

Scores are corrected for age, gender and education. P = differences between patient groups; values refer to the Mann-Whitney U-test. WCST, global score calculation = [N used cards-(completed categories*10)], higher scores mean worse performances [23]. Fluency indices calculation = for each letter (P, F, L) or category (animals, fruits, cars) a partial index was calculated in order to correct for motor disabilities as following: [(60-seconds for reading words previously reported in 1’)/N words previously reported in 1’]; the total semantic or phonemic index was obtained by averaging the 3 semantic or phonemic partial indices, respectively. Higher scores mean worse performances [21]. Abbreviations: BADA =  “Batteria per l'Analisi del Deficit Afasico”; co =  cut-off; FBI =  frontal behavioral battery; HDRS =  Hamilton depression rating scale; PLS-cd =  primary lateral sclerosis with cognitive deficits; PLS-cu =  cognitively unimpaired primary lateral sclerosis; WCST =  Wisconsin card sorting test.

### TBSS findings

**Figure 1:**
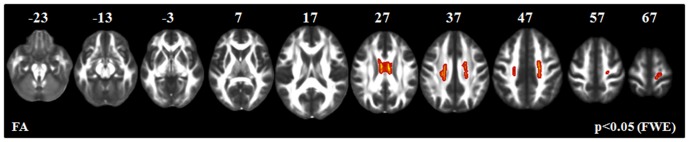
TBSS results: areas of decreased fractional anisotropy (FA, red-yellow) in PLS patients without cognitive impairment (PLS-cu) *vs.* healthy controls are displayed on a FA template in the Montreal Neurological Institute space. FWE =  family wise error.

**PLS-cu patients vs healthy controls (** **Figure 1** **).** Compared with healthy controls, PLS-cu patients showed a decreased FA in the mid-body of the CC. There was no difference in MD, radD and axD between PLS-cu patients and healthy controls.

**Figure 2:**
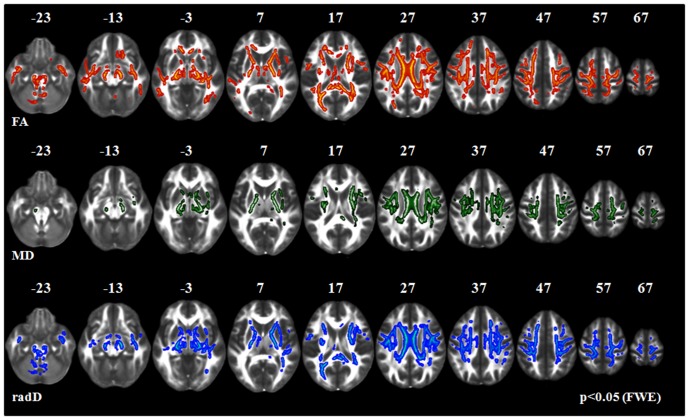
TBSS results: areas of decreased fractional anisotropy (FA, red-yellow), and increased mean (MD, green) and radial diffusivity (radD, blue) in PLS patients with cognitive deficits (PLS-cd) *vs.* healthy controls are displayed on a FA template in the Montreal Neurological Institute space. FWE =  family wise error.

**Figure 3:**
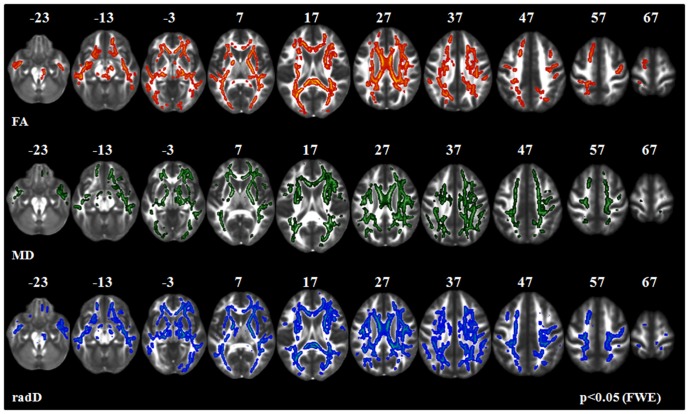
TBSS results: areas of decreased fractional anisotropy (FA, red-yellow), and increased mean (MD, green) and radial diffusivity (radD, blue) in PLS patients with cognitive deficits (PLS-cd) *vs.* PLS patients without cognitive impairment (PLS-cu) are displayed on a FA template in the Montreal Neurological Institute space. FWE =  family wise error.

**PLS-cd patients vs healthy controls (** **Figure 2** **) and PLS-cu patients (** **Figure 3** **).** Compared with healthy controls and PLS-cu patients, PLS-cd patients showed a decreased FA and increased radD of the whole CST (from the bulbar pyramids, through the posterior limb of the internal capsule, to the corona radiata and the WM surrounding the primary motor cortices), mid-body, genu and splenium of the CC, anterior limb of internal capsule, fornix, thalamic radiations, brainstem, superior and inferior longitudinal fasciculi, and parietal lobes, bilaterally (p<0.05). Compared with healthy controls, PLS-cd patients showed also a decreased FA and increased radD in the cerebellar WM, bilaterally (p<0.05). In PLS-cd patients relative to controls and PLS-cu patients, regions of increased MD were found along the CST, mid-body and splenium of the CC, superior longitudinal fasciculus, anterior limb of the internal capsule, and thalamic radiations, bilaterally (p<0.05). In PLS-cd patients relative to PLS-cu patients, further areas of increased MD were found in the inferior longitudinal fasciculus and parietal lobe, bilaterally. There was no difference in axD between PLS-cd patients and both healthy controls and PLS-cu patients.

**Figure 4:**
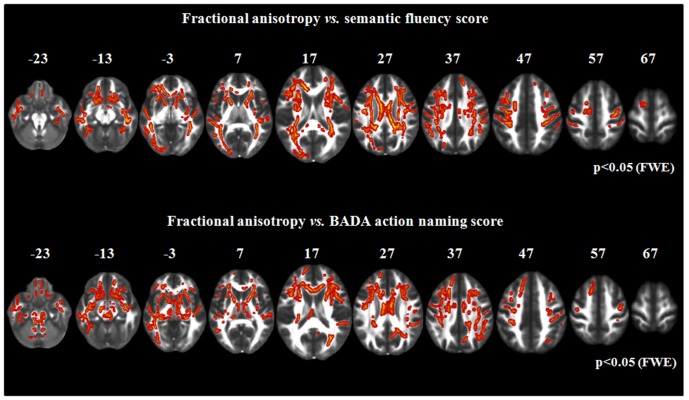
TBSS results: positive relationships between fractional anisotropy (FA, red-yellow) and patient performances at the semantic fluency test and at the “Batteria per l'Analisi del Deficit Afasico” action-naming test are displayed on a FA template in the Montreal Neurological Institute space. FWE =  family wise error.

**Relationships between WM microstructural damage and cognitive features (Figure 4).** In PLS patients the performances at semantic fluency and BADA action-naming tests were correlated to FA values of the CST, mid-body, genu and splenium of the CC, anterior limb of the internal capsule, thalamic radiations, brainstem, superior and inferior longitudinal fasciculi, frontal and parietal lobes, with a left-side predominance (p<0.05). MD, radD, and axD values did not correlate with cognitive scores.

## Discussion

This study shows that PLS-cd patients had a more severe and distributed pattern of WM damage compared with healthy controls and PLS-cu patients with similar clinical motor severity. The PLS-cd pattern of WM damage involved not only the motor cerebral structures, as in the PLS-cu cases, but also extramotor WM regions, such as the genu and splenium of the CC, prefrontal and parietal WM, fornix, anterior limb of internal capsule and thalamic radiations, bilaterally. In agreement with previous studies [10], [11], [13], [14], when compared with controls, all PLS patients showed DT MRI alterations of the mid-body of the CC.

The most common form of cognitive impairment observed in PLS-cd patients was a deficit of executive functions. Verbal fluency, problem solving and ability to maintain a set were more frequently impaired. In the PLS-cd group, however, the majority of patients showed also deficits in non-executive cognitive domains, such as verbal memory (short- and long-term memory), and language (action naming). In addition to frontal and prefrontal lobe deficits [2], [5], [6], memory and language were found to be impaired in only a few of previous PLS cohorts [5]–[8]. Poor performance in verbal and spatial memory tasks has been reported by Caselli et al.[5] In another study [6], 20 non-demented PLS patients presented with premotor cortex dysfunction (such as dynamic disintegration of the motor act and complex skilled movements) and/or executive impairment, but also with verbal memory recall and recognition deficits, as well as with grammatical deficits at a language writing test. Since the performances of verbal fluency could be misinterpreted due to patient dysarthria or spasticity, several studies investigated writing abilities in PLS patients [6]–[8]. Zago et al. [8] observed that, although all patients were able to detect and copy a set of letters, 88% of cases made a number of spelling errors (in terms of deletions, transposition, additions, and phoneme substitution) during a composing task and a dictation of words, non-words and phrases. Together with previous findings, our report suggests that the current criteria for cognitive impairment in ALS [32], which are centered on executive dysfunction, may not be optimal to identify the entire spectrum of cognitive deficits in PLS.

Consistent with the cognitive profile of abnormalities, TBSS findings showed that tissue damage in PLS-cd patients extends beyond the motor system. These findings are in keeping with pathological studies showing that ubiquitin inclusions in PLS occurr in the frontotemporal cortex [33], [34], and that phosphorilated TDP-43 immunohistochemistry reveals the presence of many positively stained neuronal cytoplasmic inclusions as well as dystrophic neuritis/neuropil threads in the frontotemporal cortex and subcortical nuclei of these patients [33]. Structural MRI studies of PLS patients showed atrophy extending into the parietal and occipital regions in addition to a pronounced tissue loss in the precentral gyrus [35], [36]. In a previous DT MRI study, the thalamus, fornix and splenium of the CC were found to be damaged in 12 non-demented PLS patients [11], [13], [14]. However, previous structural and DT MRI studies did not take into account the patient cognitive status. Only one study so far [37] has investigated the association between cognitive impairment in PLS patients cerebral hemodynamic changes. The pattern observed in four PLS cases with cognitive deficits was consistent with a frontal lobar dysfunction [37].

Extramotor WM damage may contribute to the cognitive performances of PLS patients, as supported by the relationships we observed between patient performance at executive and language tests and WM damage. Such a relationship has been also previously detected in ALS patients [38]. Perseverations, observed in our patients using the WCST, are thought to be related to damage to WM connections located in the ventral frontal regions [39]. In ALS patients, performances at the executive tests were related to DT abnormalities of the CC and major frontal connections, such as the inferior longitudinal fasciculus, fronto-occipital fasciculus, and the uncinate fasciculus [38]. Verbal fluency was found to be related to the DT MRI metrics of the left cingulum, while memory recall with those of fornix [38]. In previous studies [5], [6], memory deficits of PLS patients have been interpreted as secondary to executive disturbances (related to a frontal damage) rather than being the outcome of a direct damage to the memory storage. This is likely to be the case for only one of the two patients who experienced memory deficits in the current study, since this patient’s performance was suggestive of poor verbal encoding (which requires executive/attentive strategies) rather than deficit of pure recall. The involvement of the fornix and medial temporal WM regions in PLS-cd patients may have contributed to memory deficits in our patients. The impairment in lexical access (i.e., deficits in confrontation naming) found in six of our PLS-cd patients could be related to damage to the temporo-parietal parts of the superior longitudinal fasciculus and the inferior longitudinal fasciculus, as supported by the relationship observed between action-naming and WM abnormalities and as previously suggested in patients with primary progressive aphasia [40]. Finally, PLS-cd patients showed microstructural alterations of the cerebellar WM compared with controls. The cerebellum integrates sensory inputs to elicit precise motor control [41]. It plays also important roles across a range of cognitive and emotional functions [41]. Evidence for involvement of the cerebellum in ALS comes from several neuropathological reports, showing ubiquitinated forms of TDP-43 and ubiquitinated p62-positive inclusions in this structure, and imaging studies, which demonstrated cerebellar grey matter and WM abnormalities [42]. The structural damage to the cerebellum in MND may lead to an ineffective modulation of both motor and cognitive functions [42]. The investigation of the role of cerebellar structure in larger PLS population using structural and functional MRI can be of value in understanding the pathophysiology of the disease and its clinical and cognitive manifestations.

This study is not without limitations. First, when PLS patients were divided according to their cognitive status, the samples were relatively small. However, PLS is a rare condition; larger and multicenter studies are nonetheless needed to confirm our findings. Second, the criteria used for defining cognitive deficits in PLS (i.e., pathological score in one test) were less stringent compared with previous studies of PLS cases [3], [32], with this classification possibly being over-inclusive. In any case, this should have worked against finding significant differences rather than enhancing them. In addition, to date, there is no consensus among researchers on the definition of what constitutes cognitive impairment in PLS patients. Cognitive results of our PLS patients also showed that cognitive impairment in this condition is heterogeneous and involves cognitive domains other than the executive functions. As a consequence, we believe that consensus criteria for the diagnosis of cognitive impairment in ALS [32] (i.e., pathological scores in at least two distinct cognitive tests sensitive to executive functions) may not be appropriate in PLS patients. Thus, although healthy controls underwent a detailed neurological evaluation, we did not perform a formal neuropsychological assessment in these subjects.

In conclusion, our study shows that PLS can be associated with a multi-domain cognitive impairment. WM damage to interhemispheric, limbic and major associative WM tracts may be one of the structural correlates of such cognitive abnormalities.
